# Small UAS Online Audio DOA Estimation and Real-Time Identification Using Machine Learning

**DOI:** 10.3390/s22228659

**Published:** 2022-11-09

**Authors:** Alexandros Kyritsis, Rodoula Makri, Nikolaos Uzunoglu

**Affiliations:** 1 Microwaves and Fiber Optics Laboratory, School of Electrical and Computer Engineering, National Technical University of Athens (NTUA), 10682 Athens, Greece; 2 Institute of Communications and Computer Systems (ICCS), National Technical University of Athens (NTUA), 10682 Athens, Greece

**Keywords:** UAS, microphone array, DOA estimation, identification, machine learning

## Abstract

The wide range of unmanned aerial system (UAS) applications has led to a substantial increase in their numbers, giving rise to a whole new area of systems aiming at detecting and/or mitigating their potentially unauthorized activities. The majority of these proposed solutions for countering the aforementioned actions (C-UAS) include radar/RF/EO/IR/acoustic sensors, usually working in coordination. This work introduces a small UAS (sUAS) acoustic detection system based on an array of microphones, easily deployable and with moderate cost. It continuously collects audio data and enables (a) the direction of arrival (DOA) estimation of the most prominent incoming acoustic signal by implementing a straightforward algorithmic process similar to triangulation and (b) identification, i.e., confirmation that the incoming acoustic signal actually emanates from a UAS, by exploiting sound spectrograms using machine-learning (ML) techniques. Extensive outdoor experimental sessions have validated this system’s efficacy for reliable UAS detection at distances exceeding 70 m.

## 1. Introduction

The extensive use of unmanned aerial systems (UAS) in many military, leisure, and commercial applications, has initiated the development of a range of systems aimed to detect their presence and identify them. A layered approach [1,2] is generally deemed to be more effective in this effort, given that every detection and/or identification method is characterized by its respective drawbacks and limitations.

Active methods, such as radar, are better suited for long-range detection systems; electromagnetic signals travel faster and are less susceptible to propagation losses as compared to acoustic/sound mechanical waves. As a result, early warning systems rely heavily on active systems for the detection of distant objects. 

Passive methods (video, EO, IR, acoustic) are generally affected by weather conditions. The presence of clouds, fog, rain, etc., has a major impact on optical/electro-optical/infrared/thermal cameras’ ability to discern objects. Passive RF detection (“capturing” the link signal between the ground control station and the UAS) is less hindered by atmospheric phenomena but may require LOS with the target. As for acoustic signals, the sound absorption and attenuation while travelling through air sets significant constraints to the maximum possible distance of detection/identification. Moreover, the wideband nature of the acoustic signal characterized by rich harmonic content requires different techniques and approaches than the narrowband RF signal. 

The research for the acoustic detection and tracking of aerial bodies [aircraft, unmanned aerial vehicles (UAVs)] has been active for decades; in [3], utilizing the acoustical Doppler effect, the altitude and speed of a propeller-driven aircraft was estimated by processing the acoustic data from a microphone as the aircraft transited overhead at altitudes of between 250 and1500 ft (76–457 m).

A five-sensor cross array was used in [4] towards the estimation of the motion parameters of a moving ground vehicle’s broadband acoustic energy emissions: an early form of broadband passive acoustic techniques. The research was extended to turboprop and rotary-wing aircraft flight parameter estimations using both narrowband and broadband passive acoustic signal processing methods in [5].

Similarly, in [6], higher harmonics in the acoustic spectrogram of a UAV in-flight, as obtained from a ground-based microphone measurement, were shown to be useful for estimating the vehicle’s altitude, speed, and true engine revolutions per minute. 

A 16-element cross (16-X) array with 0.3 m spacing and a 4-element orthogonal (4-L) array with 10 m spacing (on the ground) and 1 m elevation were used in [7,8] to detect and track the flight of gasoline-engine and electric-engine UAVs. A key finding of this research was that, in contrast to the ground vehicles, the harmonic lines generated by the UAVs tended to be more stationary.

An acoustic array using 24 custom-made condenser microphones was implemented in [9]. This system was comprised of commercial off-the-shelf (COTS) hardware, while no data regarding the aircraft type or its flight path details were provided.

In terms of noise measurements for tactical (large) UAVs, an acoustic signature analysis can be found in [10,11], while an experimental analysis on the noise of propellers of small UAVs can be observed in [12,13]. The analyses of quad-copter sUAS acoustic signals using time-dependent frequency analysis and spectrograms are also presented in [14,15,16]; the main frequency content (characteristic band) was found to be below 3 kHz ([14]), between 1 and8 kHz ([15]) and up to 1 kHz and around 4 kHz ([16]). The analysis of the acoustic measurements showed a tonal component at the one-third octave at the frequency of 5 kHz in [17]. 

The possibility of using sound analysis as a UAS detection mechanism via linear predictive coding (LPC), the slope of the frequency spectrum, and the zero crossing rate of the acoustic signal was investigated in [18,19]. 

The preliminary results of audio fingerprinting techniques being used for sUAS identification are reported in [20], while in [21], sound detection by correlation is investigated; in these studies, measurements were conducted at small distances (a maximum distance of 1 m and 3 m, respectively). 

The greater part of the abovementioned research works relied on the post-processing of recorded audio data and/or the analysis of sound harmonic lines. In recent years, the substantial increase in modern systems’ computational power and the progress of machine learning (ML) have allowed for the use of more complex techniques and real-time data processing.

Reports of long detection ranges of light planes and UASs (varying in size) have been presented in [22,23,24]. However, these systems consist of either large or intricate components: a 16-microphone array (6 on the *x*-axis, 6 on the *y*-axis, and 4 on the *z*-axis) with an element spacing of 0.5 m ([22]); a 120-element microphone array complemented with a video camera ([23]); a tetrahedral shaped acoustic array with a color camera associated with a range-gated active (laser) imaging system ([24]).

Passive methods that employ machine-learning techniques on the visual data captured by cameras [for instance, in [25], where an attempt to distinguish birds from drones inside video frames using convolutional neural networks (CNNs) is presented], are outside the scope of this work. Similarly, ML techniques used in active methods [such as radar data processing ([26])] are not investigated.

A binary classification model that uses audio data to detect the existence of a drone was presented in [27]. Modern ML techniques of sound classification methods were employed [Gaussian mixture model (GMM), CNNs, and recurrent neural networks (RNNs)], achieving a reported maximum range of detection at 150 m. Measurements were obtained using a single microphone and, thus, did not allow for DOA estimation. Similarly, in [28], the use of CNNs, RNNs, and convolutional recurrent neural networks (CRNNs) was investigated; data samples were collected using a smartphone’s embedded microphone—no detection distance was reported. 

A real-time detection system is presented in [29]. FFT was performed on the sampled data and two methods for detection were evaluated: plotted image machine learning (PIL) and K-nearest neighbors (KNN). The data were collected using a single microphone, with no additional data pertaining to the systems’ hardware or the detection range being made available by the authors.

A generalization of the application concept presented in [20] is introduced in [30]; an ML-based UAV warning system was used which employs support vector machines (SVMs) to “understand” and recognize the drone audio fingerprint.

The concept of monitoring UASs to detect, control, and jam amateur drones is proposed in [31] while providing a broad description of large-scale suitable architectures for the task. 

The aircraft acoustic detection (AAD) system was presented in [32]; automated detection, classification, and tracking of low-flying aircraft using a network of passive acoustic sensors were performed by autonomously powered sensor nodes, each equipped with a microphone cluster, cameras, and complementary electronics. The research was extended in [33] where the steered-response phase transform (SRP-PHAT) was investigated and reported the systems’ ability to detect small UASs at distances of 294 m. Following these studies, [34] presented the drone acoustic detection system (DADS) which was tested along with various other acoustic systems. DADS was not introduced as a solution to provide low-cost and/or high mobility characteristics and was reported to find the DOA of a Phantom 4 UAS at up to 360 m under quiet conditions. 

An extensive review of the literature on the auditory perception of UAVs is presented in [35]. It provides a catalog of systems developed for a variety of purposes, one of which was “UAS detection”. As stated in this work, “there is not even a standard performance metric used by all the works, which makes them difficult to compare”. The authors categorized the reviewed works under groups according to the number of microphones used, localization techniques, etc. 

In the present work, we propose a lightweight, low-cost, and easily deployable cross—shaped microphone array of 4 elements that performs a 2-dimensional DOA estimation of the most prominent acoustic signals and confirms whether the signal of interest emanates from a UAS or not, thus identifying the target. The DOA estimation is achieved via a comparison between the couples of incoming signals, while identification is made possible by feeding a single channel (microphone) input to a pre-trained CNN, in order to perform the real-time classification of the acoustic data, ultimately verifying that the sound pertains to a UAS. We explicitly provide estimates for the achieved and measured range of the DOA estimation and detection, unlike many of the existing works. Exploiting the cardioid polar pattern of COTS microphones and the proposal of a deep neural network (the CNN of specific architecture) are two more contributions of the present work.

The rest of this article is organized as follows. Section 2 presents the hardware equipment used for the system implementation, gives an overview of the proposed system, introduces the fundamental principles behind the DOA estimation—identification (ML) algorithmic processes, and lays out the systems’ testing method. In Section 3, the results of the experimental sessions are presented and thoroughly discussed. Finally, Section 4 includes concluding remarks on the system’s effectiveness and highlights the possible future enhancements of the proposed system.

## 2. Materials and Methods

### 2.1. Hardware and Experimental Setup—System Overview

**Figure 1:**
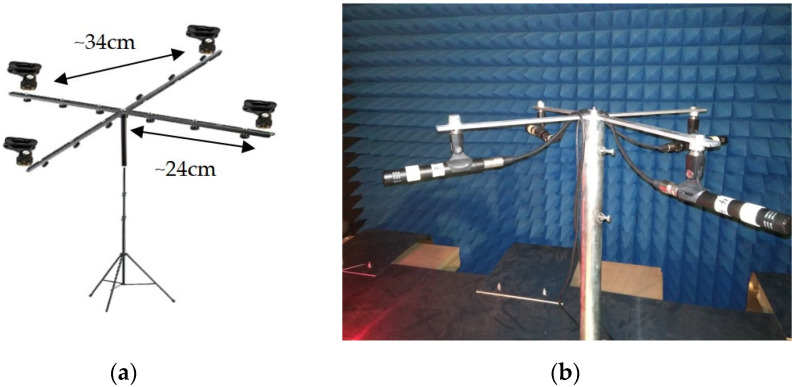
The proposed microphone array (**a**) schematic view and (**b**) hardware implementation inside an anechoic chamber.

The proposed UAS acoustic detection system is comprised of a 4-element, cross- shaped microphone array that uses an external sound card to connect to a laptop computer for data acquisition and signal data processing. An overview of the array is given in Figure 1; the microphone holding clamps were fixed on the edges of two aluminum bars bolted together perpendicularly. A metal rod was attached to the center of the bars to allow for easy mounting on larger poles, tripods, or other supporting accessories whenever additional elevation of the array was required.

The hardware specifications of the main hardware equipment used in the experimental setup is provided in Table 1.

**Figure 2:**
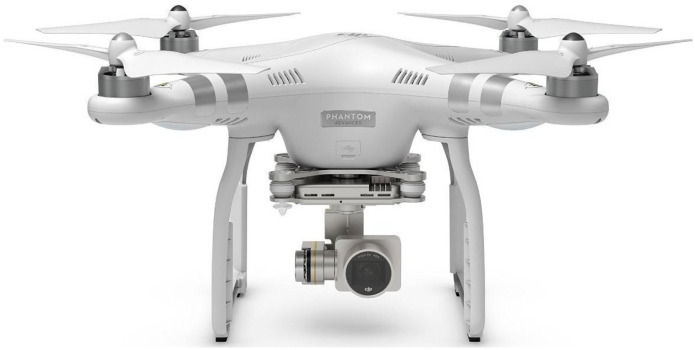
The DJI Phantom 3 Advanced sUAS used in this work.

The sUAS of choice for our work was the DJI Phantom 3 Advanced (Figure 2), a popular commercial sUAS, universally used by amateurs for recreational and semi-professional activities. 

**Figure 3:**
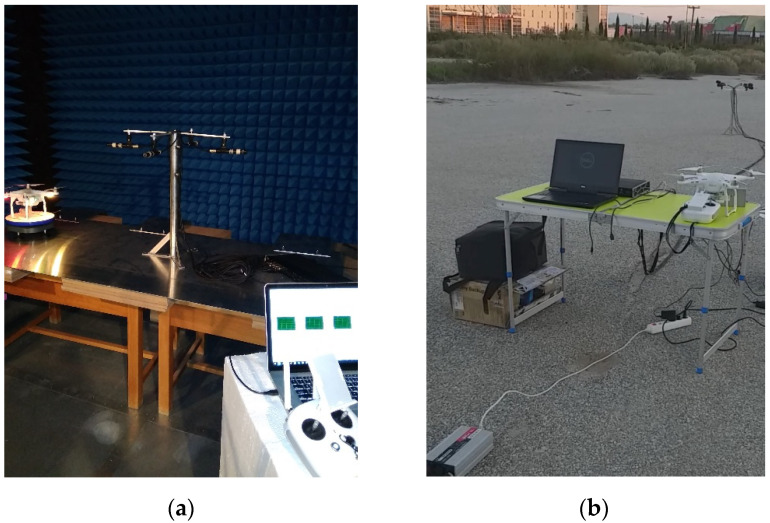
The experimental session during (**a**) indoor and (**b**) outdoor measurements.

Figure 3a illustrates the setup used during the indoor experimental sessions; they included preliminary data acquisition and microphone calibration tests during the system software development. Outdoor live measurements were conducted using the setup presented in Figure 3b.

### 2.2. Acoustic Singal Direction of Arrival Estimation

**Figure 4:**
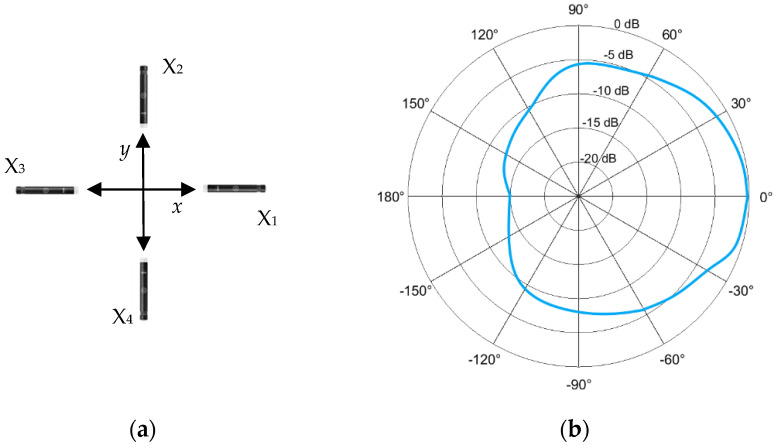
Schematics of the microphones used in this work: (**a**) Reference outline of the acoustic array; (**b**) Measured polar pattern of each microphone’s response over frequencies 1–2 kHz (comprehensive information in owner’s manual available on www.akg.com [36], accessed on 30 September 2022).

We consider the setup shown in Figure 4a, where the microphones (elements) of the array are placed in a cross-shape. Figure 4b shows a representation of the measured polar diagram of the microphone response for the frequency range 1–2 kHz. It follows a cardioid pattern, i.e., the microphone is most sensitive to sounds arriving from in front of it while picking up much less of the sounds arriving from the sides or rear. We found that the precise positioning of the microphones had an insignificant impact on the method’s effectiveness. Bearing in mind that the main objective was to seek an incoming “target-UAS” as far away as possible, the microphones were placed horizontally and slightly tilted upwards in order to take advantage of the microphone’s response at 0° (0 dB).

**Figure 5:**
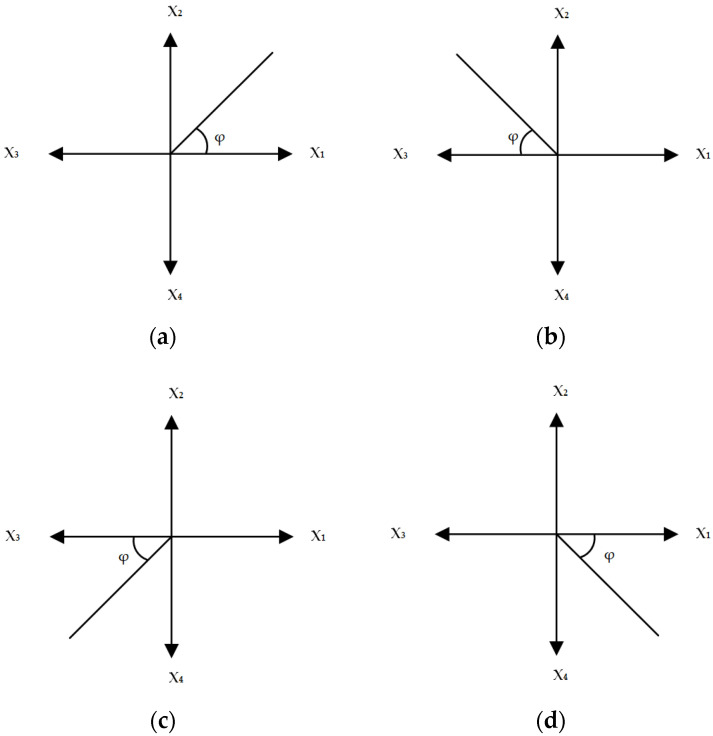
Incoming signal from (**a**) 1st, (**b**) 2nd, (**c**) 3rd, (**d**) 4th quadrant.

Let $x_{n}$ (n=1,2,3,4) represent the acoustic signal received by the microphone n. For a single microphone the polar pattern can be described by a straightforward function:

(1) $$x_{1}\left(\varphi \right)=A+B\cos\varphi \;$$

where $x_{1}$ represents the microphone response to an incident signal arriving from angle φ (as shown in Figure 5) and A, B are constants. 

Using the polar diagram of Figure 4b, for frequencies between 2 and 4 kHz, we calculated the constants A and B as follows:$\varphi =0\overset{\left(1\right)}{\Rightarrow }A+B=1$(point of 0 dB)(2)$\varphi =\frac{\pi }{2}\overset{\left(1\right)}{\Rightarrow }A=10^{-\frac{6}{10}}=0.25$(point of −6 dB)(3)


Thus, for the particular form of the diagram:

(4a) A=0.25

(4b) B=0.75

Considering a pair of perpendicularly placed microphones, the response of the second microphone [in a similar approach to Equation (1)], can be written as:

(5) $$x_{2}\left(\varphi \right)=A+B\sin\varphi \;$$

The ratio η between the microphone responses to the (same) signal arriving from angle φ can be calculated by Equations (1) and (5):

(6) $$\eta =\frac{A+B\cos\varphi }{A+B\sin\varphi }$$

Using basic trigonometric identities to transform cosφ and sinφ to tanφ, we find:

(7) $$\tan\varphi =\frac{q^{2}\eta +q\left(\eta -1\right)\sqrt{2\eta }}{q^{2}-{\left(\eta -1\right)}^{2}}$$

or

(8) $$\varphi =\tan^{-1}\left(\frac{q^{2}\eta +q\left(\eta -1\right)\sqrt{2\eta }}{q^{2}-{\left(\eta -1\right)}^{2}}\right)$$

where $q=\frac{B}{A}=3$ [given by Equations (4a) and (4b)].

Fast Fourier transform (FFT) is the most computationally efficient algorithm to compute the spectral composition for a signal of finite duration, sampled at equidistant points along the time axis. Fourier transform has been a celebrated mathematical tool—known sometimes as harmonic analysis—in physics and engineering throughout the last two centuries. In the case of a signal with period T along the time axis, Joseph Fourier, as early as 1822, showed that an infinite set of sinusoidal signals with periods T/n (with n=1,2,3,…, and an additional fixed term) can be used to mathematically describe the signal. These sinusoidal terms constitute the spectral content of the signal with the corresponding harmonic frequencies $f_{n}=n/T,$ and usually, a finite number of them are needed to compute the involved phenomena. In the case of non-periodic signals, the spectral content is continuous since T goes to infinity. In the case of a finite duration signal of $T^{*},$ and provided that the spectral content of the signal is limited to Δf, in order to reconstruct the signal accurately, one needs to sample and measure the signal every $\Delta t=1/\left(2\Delta f\right)$ and at least $N=T^{*}/\Delta t$ samples are needed. The FFT algorithm computes at N discrete points for the spectral content of the finite-duration signal. 

To meet the Nyquist theorem criteria, we set the sampling rate at 10 kHz (the maximum frequency of interest is less than 3 kHz). The following steps are taken for DOA estimation:

1. Calculate ${\hat{x}}_{1},{\hat{x}}_{2},{\hat{x}}_{3},{\hat{x}}_{4}$, where ${\hat{x}}_{n}$ represents the FFT of $x_{n}$ or $F\left\{x_{1},x_{2},x_{3},x_{4}\right\}$.
2. Calculate the sum:
  (9) $$\overset{4}{\underset{n=1}{\sum }}{\hat{x}}_{n}$$
3. For the signal given by Equation (9), calculate the number of peaks that exceed a pre-determined threshold and detect their corresponding locations.
4. For every peak detection of step 3, locate the 2 highest-amplitude signals of the original signal (step 1), which determine the quadrant of the arrival of “strongest” incoming signal.
5. Using Figure 5 as a reference, calculate ratio η as follows:
  (10) $$(a)\;1\mathrm{st}\;\mathrm{quadrant}:\;\eta =\frac{\left|x_{2}\right|}{\left|x_{1}\right|}$$
  (11) $$(b)\;2\mathrm{nd}\;\mathrm{quadrant}:\;\eta =\frac{\left|x_{2}\right|}{\left|x_{3}\right|}$$
  (12) $$(c)\;3\mathrm{rd}\;\mathrm{quadrant}:\;\eta =\frac{\left|x_{4}\right|}{\left|x_{3}\right|}$$
  (13) $$(d)\;4\mathrm{th}\;\mathrm{quadrant}:\;\eta =\frac{\left|x_{4}\right|}{\left|x_{1}\right|}$$
6. Calculate $\tan\varphi^{\prime }$ using Equation (7).
7. Calculate $\varphi =\tan^{-1}(\varphi^{\prime })$ using Equation (8).

The resulting (azimuth) angle Φ is calculated as follows:

(14) (a) 1st quadrant: Φ=φ

(15) $$(b)\;2\mathrm{nd}\;\mathrm{quadrant}:\;\Phi =180^{{}^{\circ}}-\varphi$$

(16) $$(c)\;3\mathrm{rd}\;\mathrm{quadrant}:\;\Phi =180^{{}^{\circ}}+\varphi$$

(17) $$(d)\;4\mathrm{th}\;\mathrm{quadrant}:\;\Phi =360^{{}^{\circ}}-\varphi$$

8. Repeat steps 4 through 8 for every peak found in step 3, to ensure that an averaged estimate of the DOA (quadrant of the dominant signal) is calculated.

The result of the abovementioned algorithmic process provides a quadrant and angle estimation, updated every second. As suggested by Equations (10)–(13), an inherent characteristic of this method is that it relies only on the amplitude of the FFT peaks and not on their position (frequency bin).

### 2.3. Identification Using Machine Learning Techniques

Traditional signal processing requires that the input signal is manually pre-processed\examined in order to discover certain features of interest useful to a specific task (e.g., signal pattern recognition, speech detection in arbitrary audio data). The machine-learning approach automates and simplifies this process significantly by converting the challenge from an audio signal feature recognition problem to an image classification one. Our implementation utilizes the basic principles demonstrated in [37] but is appropriately modified and extended toward our goal of identifying UAS during flight. 

We began by defining the categories under which sound samples needed to be collected and assigned corresponding labels, namely, “Aircraft”, “CH_47”, “Drone”, and “UH-1H”. In order for the system to be able to discriminate between the UAS (“Drone”) sound and other aircraft, we grouped the remaining 3 categories under the label “Other”; subsequently, the problem could be addressed as a binary classification one. The audio samples of the data set were then divided into training, validation, and test sets. 

**Figure 6:**
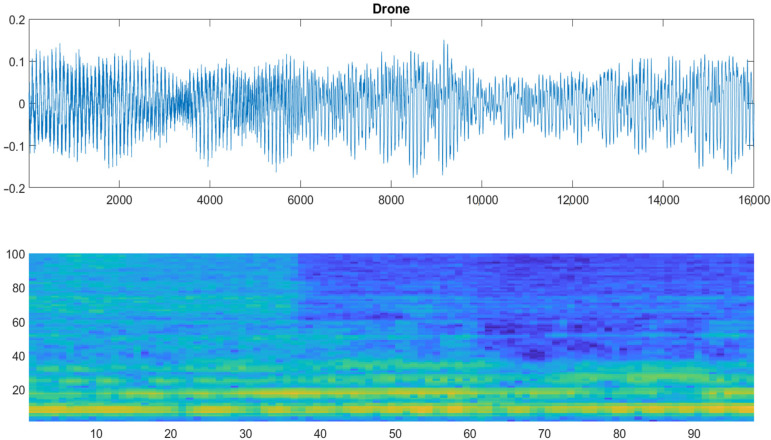
Example of an acoustic waveform (**top**) and its corresponding spectrogram (**bottom**). Spectrograms provide a visual representation of high (yellow color) against low (blue color) power signals.

The raw acoustic signal underwent a time-frequency transformation, ultimately producing audio spectrograms; a 2D image representation of the data spectrum of frequencies was produced. Auditory spectrograms of the 1-s-long audio clips were calculated using a 25 ms sliding window (frame) with a 10 ms step between the frames. Figure 6 presents an example of the time—frequency transformation for an audio file randomly extracted from the training set; the acoustic waveform is plotted in the form of samples (*x*-axis) against amplitude (*y*-axis), whereas the spectrogram is plotted as time steps (*x*-axis) against (*y*-axis) the number of filters corresponding to the frequency (100 in our case).

Since audio samples were collected from real-life environments with actual airborne vehicles being recorded during flight, the need to artificially add background noise was eliminated. However, the limited number of flights resulted in a modest number of collected audio samples; this issue was addressed using a common data augmentation method that arbitrarily increased/decreased the volume of the sound clips by up to 10% and translated them in time up to 10 times the steps (time equivalent of 0.1 s).

**Figure 7:**
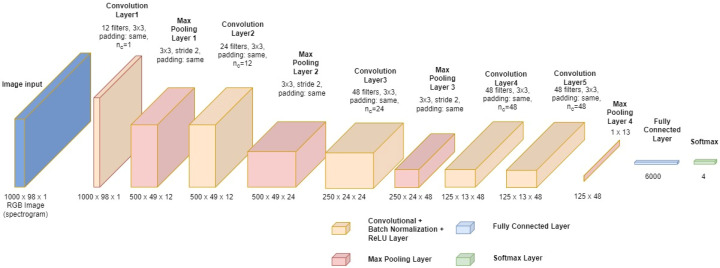
The CNN model used in the proposed system.

Figure 7 presents the architecture of the CNN used in the proposed system. It consists of 5 convolutional layers with 3 × 3 kernels, the same padding, and a gradually increasing number of filters ranging from a minimum of 12 to a maximum of 48. The model’s invariance to the local translation of input features was achieved by adding 4 max pooling layers before the final fully connected layer and, ultimately, the activation function (Softmax).

To update the network weights iteratively based on the training data, the Adam optimizer was used for training alongside a mini batch size of 8 on an Intel^®^ i7 8750H with a 6 GB NVIDIA GeForce GTX 1060. 

A total of more than 380 raw sound samples were collected, with care being taken so that the number of samples were as close as possible to be evenly distributed among the categories and avoid a training bias. The training results are presented in Table 2.

### 2.4. Testing Methods for the Proposed System

In order to verify the systems’ efficiency for estimating the DOA of UAS sound and identifying its origin, the following procedures were employed:

- The microphone array was calibrated inside an anechoic chamber to ensure that the signal captured by every element received identical amplification levels from the sound card. If the signal amplitude from a single microphone was measured -erroneously higher, an unwanted bias (possibly caused by the unintentional displacement of the analog gain knobs) would be added to the DOA estimation calculations. Calibration minimized the probability of this occurrence.
- The system was deployed in a suitable open area, and the DJI Phantom was flown in random patterns. The flight distance was monitored to evaluate the maximum range inside which the DOA estimation application resulted in the steady quadrant and angle measurements.
- Using a location that allowed for the safe observation of aircraft flights and UAS use, the system was deployed, and audio data samples were collected. After acquiring sufficient samples (during several days of trials due to the random flight schedule of the aircraft), the CNN was trained, and the identification application was tested against the real-time flights of aircraft and UAS simultaneously. Ground-truth data for the aircraft flights were not available, and only rough estimates could be made.

## 3. Results and Discussion

The proposed system was tested by conducting numerous real-time experimental sessions. Rural, suburban, and urban locations were used to investigate its performance for DOA estimation under different environments (in terms of noise clutter, obstacles, foliage, etc.). A similar approach for testing the identification algorithm was not an option, given the requirement for proximity to a helipad or airport. The following subsections describe the procedures that were followed and discuss the results of each experimental method.

### 3.1. DOA Estimation

**Figure 8:**
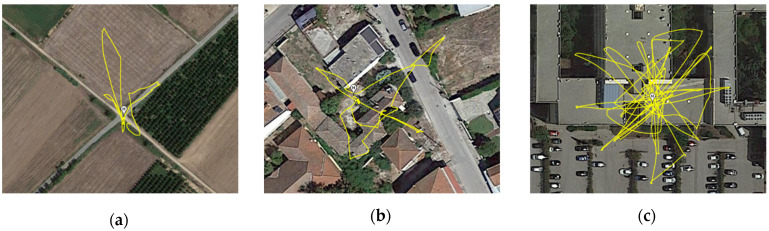
The three different types of locations used for testing: (**a**) rural, (**b**) suburban, and (**c**) urban.

Sound waves are mechanical waves and, as such, are particularly susceptible to attenuation due to propagation while travelling through the air. Apart from the distance travelled, the amount of energy (sound level) received by a microphone depends on a multitude of factors set by the environmental parameters. In order to determine the maximum range inside which the DOA estimation algorithm could provide constant and true positive indications of the quadrant, the types of locations presented in Figure 8 were chosen: (a) an open, quiet rural area with mixed foliage—no surrounding buildings or significant noise clutter; (b) a suburban area inside a small village, characterized by low buildings, moderate noise clutter, and low foliage; (c) an urban space with large buildings and significant noise clutter. At this point, it should be noted, that measurements in the urban environment were performed on a building rooftop and the reverberation/multi-path propagation of sound effects were not measured or taken into a specific account.

**Figure 9:**
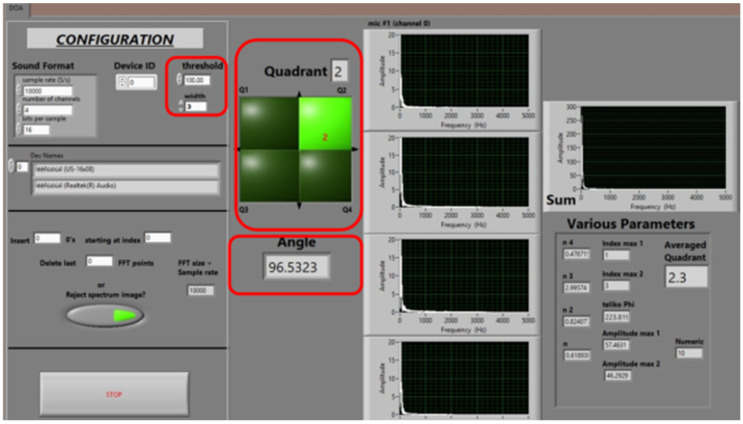
Interface of the DOA estimation application. Quadrant estimation (highlighted in green color) is updated every second. The system’s main parameters and output indicators are included in red boxes.

Next, the UAS was flown in random patterns around the area (marked with lines in Figure 8), its DOA estimation being monitored via the control interface shown in Figure 9. 

To avoid instantaneous signal peaks caused by wind gusts and surrounding noise disturbances, microphone windscreens were used, and a threshold parameter was introduced, under which all sound peaks were not taken into account during calculations. Similarly, the peak width (in FFT points) was considered, and “narrow” peaks (the corresponding parameter was set to “3”—a value empirically determined while observing the relevant graphs while no UAS was present) were omitted as spurious. The quadrant and angle indications were updated every second. 

**Figure 10:**
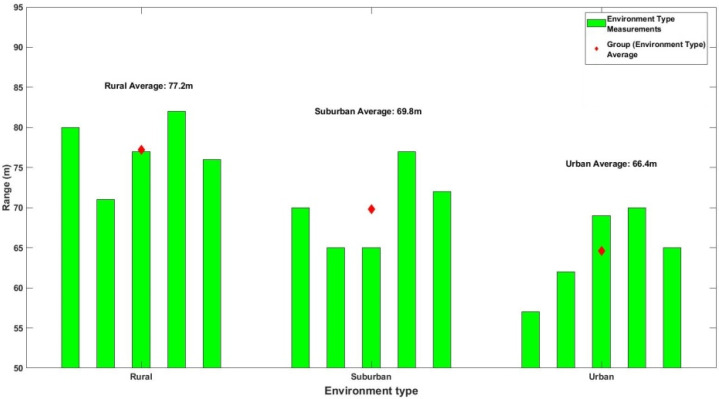
Maximum detection range for each environment type.

As the UAS moved away from the take-off home point, the distance from the microphone array increased, and the sound levels received by each element decreased. The interface of Figure 9 was monitored in order to keep track of the quadrant indication. Up to a distance, the quadrant DOA estimation remains constant, and no false alarms were detected (no abrupt changes of indication inconsequent to the UAS’s actual flight path). To determine this distance, five runs were performed on each environment type in order to calculate the average maximum distance that the DOA could be unambiguously discovered. Figure 10 presents the corresponding results.

As intuitively expected, the obstacle-free, open space of the rural environment allowed for unhindered sound propagation, resulting in DOA estimation at greater distances. On the contrary, inside the urban areas, multi-path propagation due to tall buildings and significant noise clutter had a negative impact on the system’s ability to maintain a false alarm rate of zero. Note that in every scenario, the DOA angle estimation seldom exhibited minor unpredictable fluctuations of approximately ±5°.

Table 3 summarizes the results of the abovementioned measurements. Considering the limitations of the hardware equipment used and the different environment scenarios examined, the system’s DOA estimation capability at a range of 70.53 m is confirmed.

### 3.2. Real-Time Identification Using Machine Learning

**Figure 11:**
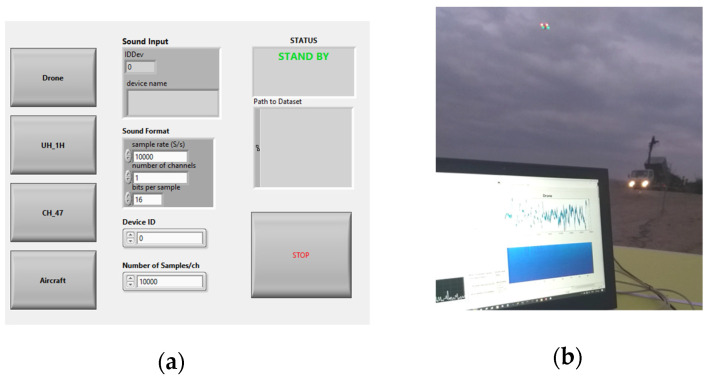
Application interfaces for (**a**) audio sample collection for CNN training and (**b**) identification of targets. Notice how the system detects the UAS’s presence, even when a heavy crane truck is working in the vicinity.

The system’s aim is to identify UAS flights; thus, discriminating incoming sounds from different airborne sources, such as helicopters and aircraft, is of key importance. Towards this goal, audio data samples were collected for training and were labeled under the categories pointed out in Section 2.3. Local airspace was observed, and every time an aircraft fly-by took place, a corresponding audio clip was recorded. UAS sound sampling was performed under less restrictive conditions because it could be flown at will—unlike aircraft and helicopter flights. Moreover, there was no need to collect samples of background noise since the data were recorded live from the actual aircraft flights that unavoidably contained background noise. Figure 11a shows the interface used for the sound sample collection, and Figure 11b demonstrates a snapshot of the outdoor measurements. The area of choice for the system deployment was located well above 150 m away from the airport/helipad.

The system’s performance was evaluated using two methods: (a) a non-binary classification, where the algorithm needed to “decide” whether the target was an “Aircraft”, “CH_47”, “UH_1H”, or “Drone”, and (b) a binary classification, which narrowed down the choices of classification to “Drone” or “Other”. 

For each of the above cases, the network had to be trained using different parameters and dataset manipulation (outlined in Section 2.3). Therefore, the system was tested during separate sessions, and different numbers of flights were monitored. Table 4 summarizes the number of flights monitored (“runs”) for each method. 

During the binary classification, due to restrictions of flight availability, the main aim was to measure the system’s performance against other airborne vehicles and not other “noise sources” in general. For example, a possible test run could be the “Drone” against the “Aircraft”/“UH_1H”/“CH_47” individually, but that would provide a very limited set of samples (e.g., 16 test samples of “Aircraft” flights against “Drone” flights). This explains the fact that the UAS detection technique’s binary classification performance was not evaluated using audio signals from other types of classes that were not “Aircraft”, “UH_1H”, or “CH_47”. 

**Figure 12:**
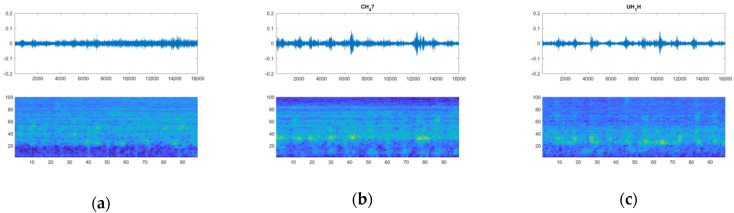
Examples of the waveform/spectrogram running plots, returning identification matches of (**a**) arbitrary sound (no match), (**b**) CH-47, and (**c**) UH-1H flights.

Two lists were completed and used for comparing the results: “True Class”, in which the actual type of the vehicle flying at any given moment was noted, and “Predicted Class”, which recorded the system-predicted category (label). Not every incoming signal produced a predicted label result; appropriate thresholds were set for the prediction probability, and the number of consecutive frames predicting the same label was needed in order to affirmatively report a detection. Whenever these criteria were not met, the system continued to buffer/unbuffer sound frames without indicating results. Figure 12 shows examples of the aforementioned process: an arbitrary sound—of substantial amplitude—produces no detection result (Figure 12a), while helicopter sound waveforms, otherwise indistinguishable, are labeled under their respective categories (Figure 12b,c). Examples of UAS (“Drone”) detection can be found in Figure 6 and Figure 11b.

**Figure 13:**
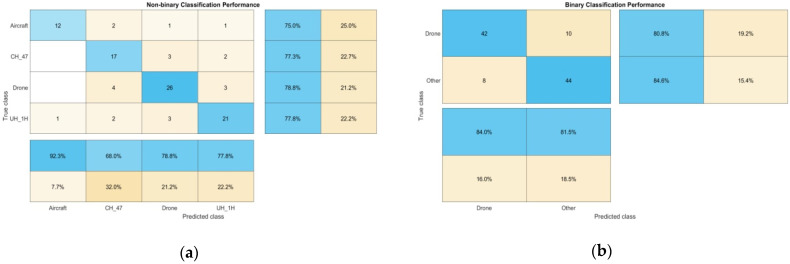
Confusion charts presenting the system’s performance over live measurements, during (**a**) non-binary and (**b**) binary classification.

Figure 13 presents confusion charts that illustrate the effectiveness of the proposed system. Incorrect predictions, represented by off-diagonal elements, are kept to a minimum and suggest a possible association between the number of aircraft rotors/propellers and the resulting predicted label; for example, the CNN was more likely to “mistake” a 2-rotor-3-winged CH-47 helicopter for a 4-rotor-2-winged DJI quadcopter (21.2%). Similarly, the 2-rotor-2-winged UH-1H was rarely mispredicted, being a 3-rotor-4-winged aircraft (7.7%). Correctly classified observations represented by diagonal elements reveal a high degree of confidence in predicting UAS presence under both scenarios (78.8% for the non-binary and 84% for the binary classification).

It should be noted that during the measurements, the aircraft number of flights, flight paths, distance, speed, and similar parameters could not be controlled. This fact proved the experiments to be more realistic but had a negative impact on the ability to collect abundant samples and attain higher precision measurements during live detection. The overall performance metrics are presented in Table 5. 

## 4. Conclusions

This work introduced a 4-element microphone array system capable of performing a dual task: estimating the position of origin in 2D and identifying the sounds emitted from a UAS. The system was built with COTS components, maintaining a low complexity, high mobility, and reliable performance.

A straightforward algorithmic process was presented for the DOA estimation. Multiple locations with diverse characteristics in terms of the clutter were used during testing to estimate the system’s ability to successfully determine the quadrant and angle of the most prominent incoming acoustic signal. Extensive outdoor testing confirmed the detection range for UAS flights at distances that exceeded 70 m.

In order to identify the sound source and decide whether it originated from a UAS, a CNN of five layers was trained using spectrograms of the acquired sound samples from real aircraft and UAS flights. The feasibility of extending the detection beyond the typical binary classification problem (“Drone” or “Other” decision) was investigated. The proposed methods resulted in a precision of 0.78201 and 0.82717 for the non-binary and binary classifications, respectively. Since flight distances of the aircraft could not be measured, the determination of the maximum identification range was impossible; as an alternative metric, the minimum distance between the array location and the airport/helipad of approximately 150 m was considered.

Overall, the system was able to provide the user with a broader awareness of the surrounding airspace since it was trained to distinguish between flying vehicles of different types and cue in on their direction.

Future work will be focused on eliminating the fluctuations of the DOA angle indication during real-time experiments and investigating the necessary requirements in order to perform range and elevation estimations. For the identification part of this research, possible future directions include the collection of more (and even more diverse) sound samples for the CNN training, verifying that the system is able to detect other types of UAS, examining any possible overfitting issues due to the environment the samples were captured in, experimenting with different NN architectures, exploring alternatives for the spectrograms of sound time-frequency representations, and the sample acquisition by four microphones simultaneously so that larger datasets may be built in less time.

## Figures and Tables

**Table 1:** Technical specifications of the hardware equipment used.

Hardware Type	Manufacturer/Model	Specifications
Microphones	AKG P170 General Purpose Instrumental Microphones	Capsule: 1/2” true condenser
		Frequency Response: 20 Hz to 20 kHz
		Sensitivity: 15 mV/Pa (−36.5 dBV)
Cables	Proel CHL-250 LU10	Length: 10 m
		Conductor resistance: 85 Ohm/km
		Diameter: 6.15 ± 0.2 mm
		Connection: XLR
Sound Card	TASCAM US-16x08	Sampling frequencies: 44.1, 48, 88.2, 96 kHz
	USB 2.0 Audio Interface/	Quantization bit depth: 16/24-bit
	Microphone Preamp	Phantom power: +48 V

**Table 2:** CNNs training performance results.

Metric	Result
CPU-time (for single image prediction)	3.55 ms
Training Error	0.57143%
Validation Error	3.3333%
Precision	0.875
Recall	0.88095
F1 Score	0.87797

**Table 3:** Results summary of the outdoor DOA estimation experiments.

Environment Type	Type Average	Overall Average (m)
Rural	77.2	70.53 m
Suburban	69.8	
Urban	64.6	

**Table 4:** Summary of the real-time test runs performed.

	Non-Binary Classification	Binary Classification
	Number of Flights	
Aircraft	16	52 (“Other”)
CH_47	22	
UH_1H	27	
Drone	33	52

**Table 5:** Performance summary for the two methods.

	Non-Binary Classification	Binary Classification
Overall precision	0.79218	0.82741
Overall recall	0.7721	0.82692
F1 Score	0.78201	0.82717

## Data Availability

Not applicable.
